# Navigating the healthcare system in Nairobi City County: perspectives and experiences in the utilization of oral healthcare by caregivers of children with HIV/AIDS

**DOI:** 10.1186/s12913-022-08260-3

**Published:** 2022-07-29

**Authors:** Mary Atieno Masiga, Simiyu Wandibba

**Affiliations:** 1 Department of Dental Sciences, Faculty of Health Sciences, University of Nairobi, P.O Box 48792-00100, Nairobi, Kenya; 2 Institute of Anthropology, Gender & African Studies, University of Nairobi, Uhuru Gardens, P.O. Box 474-00517, Nairobi, Kenya

**Keywords:** Children with HIV/AIDS, Female caregivers, Oral healthcare utilization, Healthcare system, Nairobi City County

## Abstract

**Background:**

The healthcare system in any republic can cause inequalities in health outcomes if they do not take into account the needs of deprived populations. Children with HIV/AIDS are known to have a high vulnerability to oral diseases; yet, they continue to face limitations in the utilization of oral healthcare. While other determinants of healthcare utilization may co-exist, possible gaps in the oral healthcare system can collectively affect a vulnerable group disproportionately in the utilization of oral healthcare.

**Objective:**

To explore qualitatively, the perspectives, experiences and attributions of a cohort of caregivers of children with HIV/AIDS and their Health Care Providers (HCPs), on the utilization of oral healthcare within the structure of the oral healthcare system in Nairobi City County (NCC).

**Design:**

A cross-sectional explorative mixed methods study design of two hundred and twenty one (221) female caregivers of children with HIV/AIDS and their HCPs using a survey, Focus Group Discussions (FGDs) and In-depth Interviews (IDIs). The study setting was the HIV-Care Facilities (HIV-CCFs) at three large hospitals in NCC.

**Results:**

Caregivers mainly utilized independent ‘nearby’ private dental clinics for oral healthcare services, attributing their selection to cheaper user-fees, proximal service location, and recommendations from social networks. Wait time, opening and closing hours, health workers’ attitudes and inferred opportunity costs were perceived as important quality issues in the utilization of oral healthcare.

**Conclusion:**

The oral healthcare system in NCC does not support the utilization of oral healthcare within the context of providing comprehensive healthcare for children with HIV/AIDS. Absence of ‘in-house’ oral health services at the HIV- CCFs is viewed as a defining structural barrier.

## Introduction

It is widely understood that having HIV-infection has a negative impact on oral health. While the prevalence of oro-facial manifestations is varied and may differ from region to region; consistently, these children suffer from a high prevalence of dental caries, particularly those from socioeconomically disadvantaged backgrounds who are already a high risk group for caries acquisition and poor dental attendance. In Kenya, studies carried out among children and adolescents with HIV/AIDS report the prevalence of dental caries to range from 65–84.4% [1, 2], while that of gingivitis is 86.5% [3]. Additionally, there is strong indication of unmet oral health needs and low uptake of dental healthcare among the children. Masiga et al. [1]. reported that 81% of children with HIV/AIDs had not utilized any form of oral healthcare, even when dental caries experience was associated with reduced quality of life [4]. The barriers to the utilization of oral healthcare were unclear in many of these reports. The latest HIV-Estimates Report in Kenya indicates that out of the total number of people living with HIV in 2017, 105,213 (6%), were children 0–14 years of age [5]. NCC had the 4th highest count at 8, 137 children, making them a key population group of vulnerable children in the County.

The perceived barriers of access to healthcare are typically identified as financial, cognitive and structural. Specifically, structural factors are defined as relating mostly to service availability and accessibility [6]. These factors are interrelated to components of an individual’s cost–benefit analysis in utilizing healthcare such as; distance to the health facility, financial and opportunity costs of travel, quality of care such as waiting time, attitude of providers, and ease of accessibility [7]. Additional hidden barriers may also exist, such as, knowledge about local healthcare services, non-physician gatekeeper fees (informal charges) and fear of medical care [8]. Many countries in Africa, Asia and Latin America have a shortfall of oral health personnel, and the capacity of the health systems is generally limited to pain relief and emergency care with little, if any, importance given to preventive or restorative dental care [9]. Kenya, for example, has approximately 1000 registered dentists, with a current dentist to population ratio of 1: 42,000 [10], and roughly 100 dental specialists. Most public health facilities in Kenya do not have adequate physical infrastructure for dental clinics. It is further estimated that only 1 out of 10 Kenyans has a dental health insurance cover but even then, most insurance companies including the National Social Insurance (NHIF) list dental coverage at a paltry Ksh50,000 (USD 345) annually for an average family of four (4) members [11]. Individual financing for healthcare mainly comes from out-of-pocket user fees. Additional constraints in oral healthcare delivery relate to budgetary allocation from the exchequer; for instance, in the 2014/2015 financial year, the oral health department received an operational budget of 392,400 (USD 3387) (cited) [10]_._ This is a gross underestimate of the country’s oral health needs. With these factors considered, accessibility to oral healthcare is beyond the reach of most Kenyans.

That the poor quality of health services is a major problem in many LMICs is well documented. Several accounts paint a picture of quality issues and defunct healthcare systems. In linked surveys in Rajasthan, Northern India, public health facilities were reported to open and close irregularly, absenteeism rates of doctors and nurses was found to be very high while misdiagnosis and inappropriate prescribing and treatment of patients was not uncommon [12–15]. These facilities reported very low usage of healthcare due to the low quality of care. In Uganda, East Africa, McPake et al. [16] indicated that medicines were often unavailable sometimes due to staff pilfering for use in private practice, and most health workers who had an opportunity to do so, levied informal charges. That notwithstanding, the Rajasthan survey found alternative care at private facilities to be of dubious quality. It was the convenience of health delivery in the private sector rather than proficiency of care that attracted patients away from the public sector- when they attend, people know they will find the clinic open and staffed.

In current philosophy, the user of the healthcare system is portrayed as a consumer, hence, greater responsiveness of the healthcare system is perceived as a means of attracting consumers. Today, client satisfaction is viewed as a key component of the health system’s response- the greater the responsiveness of the healthcare system to the expectations of an individual, the higher will be the level of utilization of healthcare. While the structural quality of the healthcare system relates to dimensions such as continuity of care, costs, accessibility and accommodation, patient satisfaction represents a complex mixture of perceived need, expectations and experience. Notwithstanding the widespread quality deficits reported to be common in some LMICs, high satisfaction with healthcare has been reported. Roder-Dewan et al. [17] posit that this may be due to low expectations of the populace who may lack knowledge about what constitutes good quality or they are resigned to the quality of available services.

The WHO framework commonly known as the “WHO building block”, was developed to focus attention on the need to strengthen health systems and to guide a common conceptual understanding of what constitutes a health system [18]. Six building blocks were identified as contributing to the strengthening of health systems in different ways; i) service delivery, ii) health workforce, iii) health information systems, iv)access to essential medicines, v)financing, and vi) leadership/governance. For many researchers, the framework evidently focuses on health sector actions while failing to deal with substantial and dynamic links and interactions that exist across each components; such as, the underlying social and economic determinants of the consumers, or even the problems of fragmentation and ineffective performance of public health systems in LMICs [19–21]. To this end, information is required, but gaps exist in data availability and quality. Very few developing countries are able to produce data of sufficient quality-on the demand side- to permit the monitoring of service delivery and population accessibility to essential health services. By looking through the lens of the consumers and communities who are at the very center of utilizing the healthcare system at NCC, this study seeks to provide some of this information. Though limited to a specific cohort of end-users, the information generated may be utilized in interventional methods that strengthen the processes of the healthcare system at the NCC, in particular, in the utilization of oral healthcare by caregivers of children with HIV/AIDS.

## Materials & methods

**Fig. 1:**
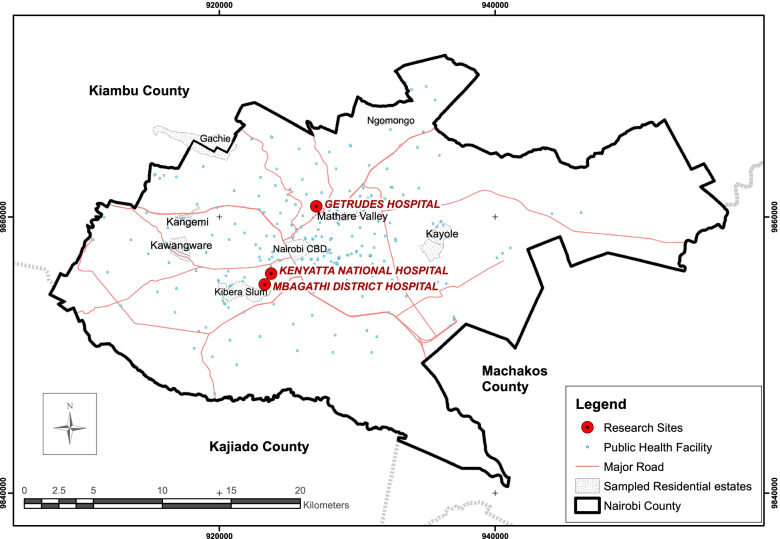
Map of NCC showing location of the research sites GCH, KNH, and MCRH. Selected public health facilities and sampled residential estates in NCC are also presented

This was a hospital-based study. The design was mixed methods, cross-sectional and exploratory. At commencement of the study in 2017, NCC recorded 496 registered health facilities at various levels of care. These are County hospitals (3), Referral hospitals (2), Health centres (71), Dispensaries (156), Medical & dental clinics (144), Maternity homes (14), Health projects (4), Nursing homes (56), VCT centres (39), others (42). During piloting of the study it was apparent that those living with HIV/AIDS source medical services at the County & Referral hospitals levels, the other smaller health facilities providing very limited healthcare. The study settings were, thus, conveniently sampled for ease of accessibility to sampled designated HIV-CCFs at these hospitals; the main referral hospital (KNH), and two county hospitals, one of which was included as a children’s hospital (GCH & MCH). Figure 1 illustrates the location of the research sites, selected public health facilities, and sampled residential areas from where the caregivers were mostly drawn, which were primarily low-income settlements.

There were two targeted cohorts as follows:

1. Female caregivers of children with HIV/AIDS attending for comprehensive healthcare at the Kenyatta National Hospital (KNH), Gertrude’s Children’s Hospital (GCH), and Mbagathi County Hospital (MCH) in NCC. These women constituted the sampling frame. The respondents for the survey were *purposively* identified and selected on a criterion basis. This involved pre-screening of all female caregivers who attended each of the HIV-CCFs within a one-week period prior to commencement of the study. The inclusion criteria were (i) female adults, 18 years and above; (ii) biological or foster mothers or kin of child/children with HIV/AIDS; (iii) caregivers of children enrolled at the respective HIV-CCFs; (iv) caregivers who had utilized oral health care for their children.
2. HCPs who dispense medical care at the HIV-CCFs, who were considered to be stakeholders due to their close interactions with the caregivers. These were *purposively* selected for those deemed to have the likelihood of enhancing the information garnered from the survey. Selection was carried out through pre-testing interviews. The HCPs who participated in the study comprised of two doctors, one clinical officer, two nurses and two social workers.

In the quantitative phase of the study, Two hundred and twenty one (221) women were recruited into a survey. A validated pre-tested questionnaire was employed to collect caregivers’ socio-demographic data, information relating to their oral health beliefs and attitudes, cultural practices in oral health, and experiences in the utilization of oral health care within the NCC healthcare system. This was followed by the qualitative phase which revisited emerging issues from the survey with a view to further shaping the respondents’ annotations and experiences. For this purpose, the focus group discussants were *alternative* caregivers who were *randomly* selected from the attending women by using the same inclusion criteria. This was done to avoid going back to the survey respondents, in order to increase the rigor of the study. Six FGDs were conducted, two at each respective hospitals, each comprising 7–8 participants. A total of 42 women participated in the FGDs, while 9 IDIs were carried out with the HCPs, three at each hospital. These numbers were considered sufficient to reach data saturation in terms of new information and/or emerging themes. The interview guides (Table 1) were developed through identifying key themes arising from the survey in line with the aims and objectives of the study. Both the FGDs and IDIs were recorded using a voice recorder and stored appropriately for transcription.

**Table 1:** FGDs & IDI Probing points

**Caregivers Focus Group Discussions**
1. Think about the times that your child attended for dental care, from which facility did you seek treatment? 2. Is the dental facility that you visit public or private? What made you select the facility that you attended? 3. Approximately how far is the dental facility from your home? 4. What transport do you usually use to reach the dental clinic? 5. How much does it usually cost you to use the transport? 6. How much time do you usually spend at the dental facility from when you arrive and leave after treatment? Does the clinic open conveniently for you to access care? 7. What is your opinion on cleanliness of the dental facility that you visit? 8. How would you describe the attitudes of the oral health providers? 9. Is there much difference between utilizing oral healthcare in private and public dental clinics? 10. Are you satisfied about the oral health services of the facility that you visit? 11. Would you voluntarily inform the dentist or other oral health provider of the HIV- status of yourself or your child? 12. What can you suggest to the county government that will help you in utilizing oral healthcare especially for your children?
**Health Care Providers In-depth Interviews**
1. What are the healthcare services provided at the HIV-CCFs? How do the patients pay for it? 2. Does the facility offer oral healthcare for children with HIV/AIDS in the same way? If not, why not? 3. What is your opinion about how caregivers of children with HIV/AIDS utilize oral healthcare? 4. What are some of the psycho-social challenges that caregivers of children with HIV/AIDS face when seeking medical care? Is it the same as when seeking dental care? 5. Do you get any form of training on oral healthcare for children with HIV/AIDS? Are you aware of the dental illnesses that these children suffer? 6. In your opinion, what are the ways that utilization of oral healthcare can be enhanced at these facilities for children with HIV/AIDS?

### Data processing and analysis

Quantitative data from the survey was analyzed using the Statistical Package for the Social Sciences (SPSS) Version 19.0. Cross-tabulation of variables was carried out using Chi Square tests and ANOVA. For qualitative data, we identified words and phrases that represented broad categories and specific themes. Coding system was used to categorize the themes according to the objectives of the study where major themes and categories resulting from the coding process were organized into thematic matrices to determine common concepts.

## Results

### Distribution of respondents by hospital category

A total of 221caregivers participated in the survey; 45% were drawn from KNH, 28% from GCH, and 27% from MCRH. The distribution of respondents across the hospital categories was done proportionately to correlate with the number of children enrolled and active-in-care at each respective hospitals.

### Socio-demographic characteristics of the respondents

The respondents were primarily biological mothers of children who are enrolled at the HIV-CCFs, and were mainly in the young to middle-aged categories. The average age of the children was 9.64 years (± 6.46 SD). Table 2 summarizes the socio-demographic characteristics of the respondents. The ages, marital status and household income levels of the respondents at the three hospital categories were not significantly different (*p* = 0.522; *p* = 0.549 and *p* > 0.05 respectively); subsequently the results are discussed collectively.

**Table 2:** Sociodemographic characteristics of respondents

Variables	Category	Frequency	Percentage
Age (years)	18–25	9	4
	26–33	60	27
	34–41	91	41
	42–49	38	17
	> 50	15	7
	Don’t know	8	4
Relationship with child	Biological mother	168	76
	Grandmother	38	17
	Aunt	11	5
	Unrelated	4	2
Marital status	Married	130	59
	Widowed	31	14
	Separated/divorced	27	12
	Single mother	33	15
Highest education level	No formal schooling	12	5
	Primary level	95	43
	Secondary level	73	33
	Tertiary level	40	18
	Don’t know	1	1
Employment status	Informal	108	49
	Formal	62	28
	Casual (menial)	13	6
	Unemployed	38	17
Household income	= < 10,000	93	42
(KES)	11,000–20.000	42	19
	21,000–30,000	22	10
	31,000–40,000	15	7
	41,000–50,000	11	5
	> 51,000	20	9
	Don’t know	18	8
Health insurance Status	No health insurance	150	68
	Employer health insurance	46	21
	Personal health insurance	18	8
	Don’t know	7	3

Source: Survey data, 2017

### The utilization of oral healthcare services

#### Reasons for choosing oral health facility

**Fig. 2:**
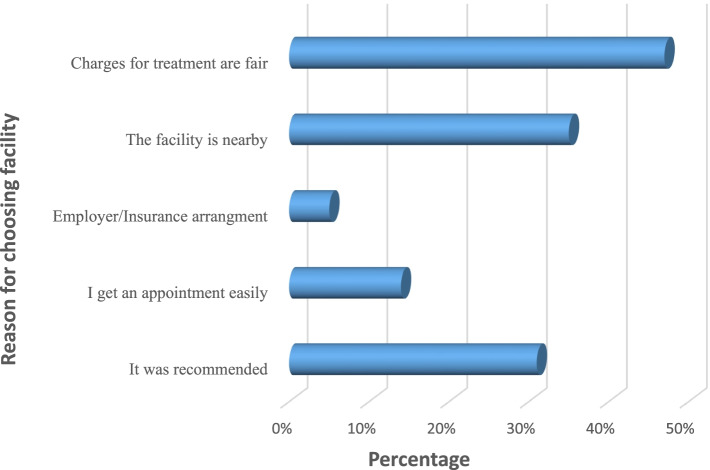
Respondents’ reasons for choosing oral health service facility. (Source: Survey data, 2017) *Percentage is more than 100% because of multiple responses

Respondent’s reasons for choosing oral health facilities were mainly centered on perception of “fair” user-fees, the proximity of the oral health provider, and recommendations from others in their social networks (Fig. 2). The lay referral network system appeared to play a fair role in influencing the caregivers’ selection of oral health providers.

#### Distance travelled to oral health facilities

The distance to service locations ranged from > 1 km to < 15 km. The modal distance was 1–5 km (Table 3). There was, however, no association between distance travelled and the utilization of oral healthcare (*p* > 0.05).

**Table 3:** Distance from home to oral health facility

Distance (km)	Frequency	Percentage
> 1 km	12	9
1 to 5 km	39	29
6 to 10 km	36	27
10 to 15 km	17	13
More than 15 km	25	19
Don’t know	4	3
**TOTAL**	133*	**100**

(Source: Survey data, 2017) *No of children who had visited the dentist

Below, are sampled views arising from the FGDs and IDI on caregivers’choice of oral health facilities:

*The dental clinics we frequently visit are nearby and a short distance from home. Some are located at the shopping centres where we go every day. We can walk there when we have toothache, get attended to quickly and go back to work without wasting a lot of time in travel. Another reason is that we don’t require fare to get there (FGD, KNH).*

Having to travel long distances is quite a challenge for our patients. We notice that this often causes them to fail in attending for their medical appointments. Many of the patients come from *Eastlands or even further. They often report that they have no fare even when appointments are spaced 3-4 months apart (Social worker, GCH).

*Eastlands-a high density area typical of where most respondents in the study resided.

#### Mode of transportation to oral health facilities

From the results, 32% respondents walked from home to the oral health providers of choice, while others used various means of transport (Table 4).

**Table 4:** Mode of transport to oral health facility

Mode	Frequency	Percentage
Walk	43	32
Buses (matatus)	48	36
Motorbikes	11	8
Matatus & Motorbikes	13	10
Private cars	8	6
Taxi	10	8
**TOTAL**	133*	**100**

(Source: Survey data, 2017) *No of children who had visited the dentist

A doctor at GCH made the following statement on cost of transportation and the inconveniences of long distances of travel*: ‘Being mainly low-income patients, the cost of transportation is a major problem for many women who come here. Some of them have to save money for several months to ensure that they have transport for their next appointment. There are also other inconveniences, for example, ‘the matatus’ drop them at the main road and then they have to use motorbikes to reach the hospital whilst carrying their children (Doctor, GCH).*

#### Categories of oral health facilities utilized

The caregivers mainly (57.1%) utilized small independently-owned private dental clinics to access oral healthcare services (Table 5).

**Table 5:** Categories of oral healthcare facilities visited

Category of facility	Frequency	Percentage
Government/public hospitals	26	19.5
Private hospitals	8	6.1
Private clinics	76	57.1
Mission/charitable hospitals	20	15.0
Herbalist	2	1.5
**TOTAL**	133*	**100**

(Source: Survey data, 2017) *No of children and caregivers who had visited the dentist

They defended their choice of oral health providers as captured in the following statement from participants in one of the FGDs.

*The small private clinics are convenient for many reasons. They are located very near to where we live and we can easily access them when we require treatment. We mostly don’t need to make an appointment. Furthermore, they are less expensive and sometimes we get a discount when we don’t have enough money; they charge between KES 100-300 ($ 0.93-2.78) for tooth extraction for children. Most of us can actually afford that (FGD, GCH).*

### Perceptions on quality of care

#### Satisfaction with clinic opening hours

The convenient opening and closing times and long working hours at the private dental clinics enabled the caregivers to access care for their children after school hours without the need to pull them out of school, unlike the public dental facilities which were said to close very early. This is how the FGD participants at MCH articulated their views:

*The clinics we visit open early and close late in the evening, sometimes up to 10.00pm, and they also open on Saturdays. This is convenient for us. Our children first go to school, then we can take them for treatment later in the evening or on the weekends. This works very well for everyone. Public dental clinics close very early, in fact; by 3.00 pm, they send you away because the clinic is closed and they will not open on weekends. (FGD, MCH).*

#### Satisfaction with waiting time

There were longer waiting times reported of up to 3–4 h at the public dental hospitals compared to an average time of 1 h or less at the private dental clinics. Respondents were concerned about the loss of working hours and consequent opportunity costs due to long waiting times at public dental facilities. Group discussants supposed the need to have a ‘friendly’ insider at the public facilities to assist in speedy access to care, as below:

*At the public dental hospitals, the waiting can be very tiresome. There are always long queues but if you know someone from the inside, they will help you to be seen quickly; otherwise, one can waste the whole day there, and in the end our work suffers. Many times we would rather put money aside and visit the private dental clinics where we get treated much faster and move on (FGD, KNH).*

Notwithstanding the long queues and extended waiting times, the public hospitals were viewed to have medical experts and specialists who enhanced the quality of healthcare. The discussants at the FGDs in KNH & MCH were in agreement as below:

*In public hospitals, you find specialists who are capable of treating any kind of serious illnesses or conditions. They are well- qualified and they know their work so they give us confidence. Some of the private clinics do not have very qualified doctors; you can easily find quacks over there (FGDs, KNH & MCRH)*

#### Satisfaction with cleanliness and maintenance

Both categories of oral health facilities (private and public) were viewed to have acceptable standards of cleanliness, although private dental clinics were rated better than the public clinics in terms of facilities. Majority (68%) of respondents reported cleanliness at the private health facilities to be ‘very good’, while public facilities ratings were mainly centered at average, as captured in the statement below:

*The dental clinics are usually quite clean. Even the public clinics are well maintained but you can see that the facilities are old, some even broken down. Of course the facilities at the private clinics are always better (FGD, MCRH).*

#### Satisfaction with attitudes of health workers

At the private dental clinics, the attitude of the health workers was termed as ‘very good’ by more than half (56%) of the respondents, good by 32%, and average and below by 12%. The ratings for public facilities were 22%, 40% and 38% respectively. Health workers in government facilities were seen as not caring enough, and did not interact sufficiently with patients. The participants in one FGD attributed this to the large number of patients visiting the public hospitals, and stated:

*The health workers at the public facilities have a rather uncaring attitude compared to the ones at the private dental clinics. They don’t give us enough time to explain anything, instead they shout and sometimes accuse us unlike in the private clinics where they are polite and handle us well. But we can also see that they are overwhelmed by the number of patients that go there (FGD, MCRH).*

#### Satisfaction with oral health services

**Fig. 3:**
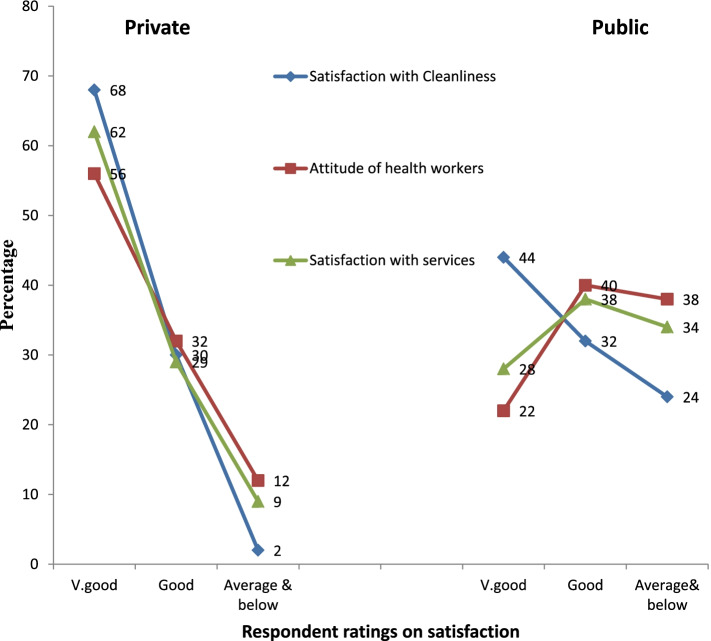
Respondent’s ratings on satisfaction with cleanliness, attitude of health workers and satisfaction with services at private and public oral health facilities. (Source: Survey data, 2017)

Satisfaction with oral health services was considered better at the private dental clinics with respondent rating of ‘very good’ at 62%, while the public dental facilities were mainly rated as average. Figure 3 illustrates high ratings of patient satisfaction at the private dental clinics in the three domains of cleanliness, health worker attitudes and service, while the ratings for the public facilities are mostly average.

Caregivers regarded the most outstanding aspects of service delivery at the private dental clinics to be the speed and efficiency with which they received services unlike the delays they experienced at public health facilities. A clinical officer at KNH attributed the long queues at public health facilities to tedious processes and bureaucracies:

*Patient go through several processes at public hospitals that just take too long. They queue to open a file, make payments, do lab tests and other investigations; then they queue again to be attended to. Often there are x-rays to be done. At the pharmacy the patients further queue for medicines. If they get stuck at one process, they can’t proceed to the next one. Frequently, they get reappointed even after spending that much time queuing (Clinical officer, KNH).*

### Perceptions on oral health & dental illnesses

According to a nurse at KNH, most patient do not give much significance to dental illnesses, and they don’t seem to regard oral health as vital to their general health. This was her statement: “*The patients here are keener on taking medications for their condition, to sustain their overall health, making oral healthcare not such a big deal to them.”*

The abstraction of the mouth from the body permeated even among the HCPs themselves. There was self-report of own deficiencies in oral health literacy. *“Even we, as health workers lack the correct information to give our patients on oral health and the proper utilization of oral healthcare. We can’t make recommendations to our clients which we ourselves are not practicing” (Counselor, KNH).*

Additionally, the protocol for patient review at the HIV-CCFs does not provide for much in terms of oral healthcare, according to this statement by a clinical officer: *“We don’t spend much time on clinical examination of the mouth and the teeth. We just check to see if patients have fungal infection (candidiasis) on the tongue. Besides that, even if they have dental caries but do not complain of pain, we don’t pay much attention. Moreover, the software that we use to review patients has a very small component of oral health examination (Clinical officer, KNH).*

Further, there are no formal oral health services provided at the HIV-CCFs, nor much participation from dentists and/or other cadre of oral health providers. A doctor at KNH stated his concern in the following testimonial:

*We require the input of the dentists and other oral health providers to sensitize our patients on oral healthcare. At KNH, we have other integrated specialized services; for example, the dermatologists come to see our patients on regular specified days. We do not get any dental consultations so we see that as a weak area (Doctor, KNH).*

#### ‘Entitlement’ to oral healthcare

As a result of being able to access *free* medical at the HIV-CCFs, caregivers were seemingly unwilling to pay out-of-pocket (OOP) expenses for oral healthcare elsewhere. They perceived an entitlement to free medical care all round, according to the following statements garnered from the HCPs:

*The patients who come to the HIV-CCFs are attended to free of charge, inclusive of consultation, lab tests and medicines. They are used to that. I believe they shy away from seeking dental services because of having to pay for these services from out of pocket (Pharmacist, KNH).*

*When our patients go to source health services elsewhere, they have to pay consultation fees and other user expenses. This applies to oral healthcare as well. A number of them can probably afford it but are unwilling because they are used to free medical care at the HIV-CCFs (Clinical officer, KNH).*

*We give every aspect of HIV-support in these clinics free of charge. However, we are not able to provide specialized treatment procedures such as X-rays or dental care, therefore, for such cases we refer the patients outside. When we say that to the women, they ask, ‘is it free’? When we tell them that it is not they tell us, ‘I will go another day’. I doubt that they go; it is obvious that they expect treatment to be free (Doctor, GCH).*

### Expectations on oral healthcare delivery

Caregivers expressed a strong desire to have government-run public dental clinics at the grassroots for easier and more convenient access to regulated oral healthcare services. There were also a call for more government subsidy for oral healthcare services.

*The dental clinics in the county government are located very far from where we live. ‘Kule mashinani hakuna mahospitali ya meno ya serikali’ (there are no county dental clinics at the grassroots). Many times when we go to the county health centres, we don’t find a dentist. We are told they only come once or twice a week (FGD, KNH).*

*At the same time, the government should make the cost of oral healthcare more affordable because many children have issues with their teeth. If you go to the public hospitals, there are so many patients seeking care and yet the cost of treatment is still too high (FGD, MCRH).*

## Discussion

The geographical inaccessibility to health facilities frequently causes patients to use different modes of transport to access formal healthcare, whereas close proximity of the health provider is beneficial in cutting down the cost of transport and the inconveniences of travelling long distances. Hausen-Muela et al*.* [22] posit that limited access to transportation and costly public transport in particular, are among challenges facing women while seeking healthcare. The caregivers in this study circumvent these challenges by mainly utilizing small independent-owned ‘nearby’ private oral health facilities, where the modal range of distance travelled is 1-5 km. While these facilities are convenient, anecdotal reports infer that they may not always be regulated in the care they provide. This weighs in on the actual quality of care accessed by the caregivers. It was interesting to note that caregivers were willing to travel longer distances for medical care than for oral healthcare, suggesting preferences in seeking healthcare for their chronic medical conditions against the preservation of oral health. Patients in low-income countries have been reported to demonstrate this passive-active health-seeking behavior for the different facets of health. Information is collected and used on choices that most improve their healthcare outcomes [23]. Perceived low quality of public primary health care often results in patients actively forgoing (“bypassing”) care at the nearest health facility and seeking proper care at a higher-level public facility or in the private sector [24, 25]

The time spent at a health facility and the forgone earnings were viewed as significant additional costs to consuming healthcare. In the caregivers’ opinion, the long hours spent in accessing oral healthcare could well negate a whole day’s worth of work. Similar findings on the demand-side factors of healthcare utilization in Ghana were reported by Russell [26] who posited that individuals may work through sickness instead of going to health providers, because of actual and time costs as well as loss of income from a day’s work for-gone, as the caregivers in this study. Could compliance in health-seeking be more easily improved in those who are not economically active since they are more likely to have time to attend for treatment? It is of course prudent to balance such views by the other far-reaching effects of low incomes.

Frequently, when patients do not feel competent to judge the technical quality of health services, they base their satisfaction of quality of care on the timeliness with which they receive services and the promptness in being attended to by the health care providers at the health facilities. Similarly, the caregivers in this study view the expediting of oral healthcare services at the private clinics as quality issues. There was also the convenience of long opening hours including the week-ends, which saved on school hours for their school-going children. Notwithstanding, the caregivers are cognizant of the enormous workload placed on health workers at the public hospitals as a result of the high influx of patients. As a component of health policy, inadequate staffing at health facilities and a poor balance of provider-health consumer ratio could result in health workers’ poor attitude towards their work [27].

This study also denotes the inordinate extent of private participation in healthcare systems. Given the increasing capacity limitations of the public sector to meet client expectations in most governments, it is inevitable that the more formal private sector will have a significant supplementary role in managing health services. Mills [28] posits that because the capacity of the government sector is limited and there is concentration of human resources in the private sector, seeking a mix of private–public provision of services can be seen as a pragmatic response. Even so, she contends that engagement of the private sector remains a topic of considerable controversy seen by some as inviting the privatization of healthcare and making it a prized commodity.

The definition of basic services to be provided and the mechanisms for financing these provisions in health service delivery is a significant aspects of an ‘unalienable right’ healthcare system. While it was clear that caregivers play out the ‘Entitlement Syndrome’, or deservedness to all privileges in healthcare, the study noted that there are no concessions within the structures of the HIV-CCFs in NCC for provision of oral healthcare services. A chasm exists that appears to have removed the mouth from the body and disenfranchised children with HIV/AIDS from accessing oral healthcare at the point where they receive *free* medical care. A previous study on this cohort reports that more than two-thirds of caregivers do not have any form of health insurance; and, majority who access oral healthcare pay from out-of-pocket expenses [29]. For low-income earners, this is a balancing act with the likelihood of regulating preferential allocation of household resources at the expense of non-urgent oral healthcare. Deficiencies in quality of care can have direct implications for access to effective healthcare. Children with HIV/AIDS in NCC have been found to consume oral healthcare poorly, and do not have a usual source of care [30].

## Conclusion

Female caregivers mainly choose small independently-owned ‘nearby’ private dental clinics to utilize oral healthcare because this provides them with ease and convenience of accessibility. This study infers that the unavailability of oral healthcare services within the confines of the HIV-CCFs, coupled with perceived unsatisfactory services at public oral healthcare facilities creates structural barriers to accessibility of regulated oral healthcare for children with HIV/AIDS within the processes of the healthcare system in NCC.

## Recommendations

The study recommends improved integration of satellite dental clinics within the oral healthcare system of NCC to enable the populace access convenient, regulated oral healthcare. This should be inclusive of basic oral health services as a component of comprehensive healthcare for children with HIV/AIDS at the HIV-CCFs.

### Bullet points

1. Improvement in oral health for vulnerable groups is an important indicator of the overall performance of an oral healthcare system.
2. Reviewing and developing oral health indicators appropriate for developing countries will advocate for the oral health of vulnerable children in LMICs.

### Limitations of the study

The data for this study was garnered from three large hospitals where the HIV-CCFs in NCC are domiciled. It is deemed that the data produced is representative and therefore, it is suggested that the information may be generalizable with caution. The decision to restrict participation to female caregivers was informed by a previous study at KNH [4] which observed that majority of caregivers (88.2%) who accompany their children to HIV- CCFs are female. Triangulation facilitated the validity of data through cross verification. It was beyond the scope of the study to implement any interventional measures.

## Data Availability

All data sets generated or analyzed during the current study are available from the corresponding author on reasonable request.

## Abbreviations

HCPs: HealthCare Providers
NCC: Nairobi City County
HIV-CCFs: HIV Comprehensive Care Facilities
GCH: Getrude’s Children Hospital
KNH: Kenyatta National Hospital
MCH: Mbagathi County Hospital
LMIC: Low & Middle Income Countries
NHIF: National Hospital Insurance Fund
OOP: Out of Pocket Payment
IAGAS: Institute of Anthropology, Gender & African Studies
UON: University of Nairobi
